# Maternal socioeconomic status and the risk of asthma among offspring

**DOI:** 10.1186/s12889-015-1357-6

**Published:** 2015-01-28

**Authors:** Maijakaisa Harju, Leea Keski-Nisula, Leena Georgiadis, Kaisa Raatikainen, Sari Räisänen, Seppo Heinonen

**Affiliations:** Department of Obstetrics and Gynecology, Kuopio University Hospital, Kuopio, Finland; University of Eastern Finland, Faculty of Health Sciences, Kuopio, Finland; Helsinki University Central Hospital, Helsinki, Finland

**Keywords:** Asthma, SES, Childhood, Pregnancy

## Abstract

**Background:**

To evaluate the association between maternal socioeconomic status (SES) during pregnancy and asthma among offspring.

**Methods:**

A retrospective observational hospital-based birth cohort study in a university-based Obstetrics and Gynecology department in Finland. A total of 40 118 women with singleton live births between 1989 and 2007 were linked with data from the register for asthma medication for their offspring (n = 2518). Pregnancy and maternal SES factors were recorded during pregnancy and labor. SES was categorized thus: upper white-collar workers (highest SES), lower white-collar workers, blue-collar workers, others (lowest SES) and cases with missing information. Logistic regression analysis was used to determine the association between maternal SES and childhood asthma.

**Results:**

We found no convincing evidence of a direct association between maternal SES and childhood asthma. Parental smoking was the clearest factor affecting asthma among children of lower white-collar workers. Differences in pregnancy and delivery characteristics were observed between the SES groups.

**Conclusions:**

Maternal socioeconomic status had no significant direct impact on the prevalence of asthma in this Finnish birth cohort. Finnish public health services appeared to offer equal quality services independently of SES.

**Trial registration:**

The study is registered in Kuopio University Hospital register (TUTKI): ID5302448.

## Background

Asthma and allergic diseases in childhood affect nearly 10% of children worldwide, the prevalence being lowest in Asian countries (2–4%) and highest in developed countries (15–20%) [1,2]. The prevalence has been increasing in recent decades, although part of the increase might be due to increased rates of reporting and awareness of the symptoms [3]. Both pre- and postnatal factors play an important role in the development of asthma [2,4,5]. Early epigenetic programming of asthma and allergy is suggested to occur in utero, where various forms of prenatal exposure such as maternal smoking, stress and anxiety, infections, infertility treatment and restricted fetal growth might affect development of the fetal immune system [3-6]. Furthermore, potential risk factors related to delivery and the neonatal period include prematurity, low birth weight, birth mode and early-life infections [1,3,4,7]. In addition, parental history of asthma, male sex and exposure to certain environmental factors have been associated with asthma in childhood [2-4]. All of these perinatal factors are linked to parental socioeconomic status (SES) and thus socioeconomic disparities may influence the burden of asthma.

Finland has had a highly developed publicly funded primary healthcare system for many decades, every pregnant woman receiving free or low-cost high-quality maternity care during pregnancy and delivery. More than 98% of all deliveries occur in publicly owned birth hospitals. Recently, Räisänen and associates showed in a large birth register study in Finland that low maternal SES strongly determined the need of cesarean delivery [8], as well as being a strong predictor of small-for-gestational-age (SGA) infants [9]. The aim of the present study was to identify perinatal differences between socioeconomic groups and to clarify their contribution to asthma among offspring by conducting an observational study using hospital-based birth register data on 40 118 women and their singleton live-born infants in 1989–2007 in Finland.

## Methods

Data were collected from the Kuopio University Hospital clinical birth database, covering 42 205 infants who were born after 23 completed weeks of gestation between 1989–2007. We excluded multiple pregnancies, stillbirths and neonatal deaths. After these exclusions, data on 40 118 women with singleton live-born infants were linked with data from the register for asthma medication for their offspring aged 1–19 years (n = 2518).

Information on maternal pre-pregnancy characteristics was based on data from self-administered questionnaires at 20 weeks of pregnancy. Midwives and public health nurses completed missing data by interviewing during visits to prenatal maternal clinics or labor wards at Kuopio University Hospital. The questionnaire covered 75 background items [10]. At the time of childbirth and during the neonatal period nurses and midwives added information on pregnancy, delivery, neonatal outcomes and complications to the database. Informed consent for this register study was given by childbearing women at the time of data collection.

Information on the need for anti-asthmatic drugs during childhood was obtained from the Drug Prescription Register of the Social Insurance Institution. Anti-asthmatic drugs were explored and categorized according to FIMEA (Finnish Medicines Agency) ATC((Anatomical Therapeutic Chemical)-groups: R03, R06, drugs for obstructive airway diseases consisting of as-needed inhaled beta2-agonists, inhaled glucocorticoids, inhaled long acting beta2-agonists and antileukotrienes [11]. In Finland, all medication is prescribed by physicians, and in the case of pediatric asthma, usually by pediatricians, and reimbursed at a rate of 72% via the National Sickness Insurance Scheme [12]. The diagnosis of asthma among offspring was evaluated in two different ways in this study: based on the doctor diagnosed asthma (set by a paediatrician using ICD code) and based on the information on over six month continuous use of anti-asthmatic drugs. Permission for the use of register data in scientific research was granted by the register-keeping organization. Information on maternal socioeconomic status was based on the mother’s occupation at the time of birth based on the EU’s classification of occupations ISCO-88(COM) [13]. At first, five SES classes were categorized: upper white-collar workers such as teachers and physicians, lower white-collar workers such as nurses and secretaries, blue-collar workers such as cooks and cleaners, “other”, which included housewives, students, and unemployed and retired people, as described in detail elsewhere [14], and entrepreneurs. Entrepreneurs were separated into a different group in our data and consisted mainly of producers of agricultural goods. Since they constituted only 2.9% of all study women, this group was included in “other”, making classification similar to that in previous Finnish studies [9,14]. Information on SES was missing in 2324 (5.8%) cases and this group was analyzed separately.

Statistical analyses were performed by using SPSS 21.0 for Windows. Pearson’s chi-square and Mann–Whitney *U* tests were used to analyze univariate data on maternal and prenatal pregnancy characteristics and SES.

Multivariate logistic regression analysis was performed to model the association between asthma among offspring, and maternal SES. Confounding risk factors were selected on the basis of univariate analysis using a cut-off p value of <0.05 for factors with known clinical importance. The multivariate analyses were adjusted for maternal asthma (no vs. yes), maternal hypertension (no vs. yes), maternal parity (0, ≥1), ART (assisted reproduction technology before the index pregnancy (no vs. yes)), parental smoking (nonsmoking parents, maternal or paternal smoking, both parents smokers), mode of delivery (vaginal vs. cesarean), child’s sex and gestational weeks at birth (<37, 37–40, ≥41 weeks). Furthermore, the children’s follow-up time was taken into account by using the child’s age at the onset of asthma or age at the end of the study among healthy children (2007). Upper white-collar workers were selected as the reference group, where the incidence of asthma was nearest to its mean prevalence. Differences were deemed to be significant if p was <0.05.

The study was approved by Ethics Research Committee of Kuopio University Hospital and the Committee gave permission for the results to be published (93//2008).

## Results

The study population covered 40 118 singleton live births, among which 2518 (6.3%) offspring had asthma during childhood. The mean age was 4.2 years at the onset of asthma and 82.3% of these children were aged more than seven years at the time of the study. Compared with controls, asthmatic children were more frequently males born by cesarean delivery, at lower gestational age and more frequently with birth weight under 2500 grams and small for gestational age (SGA). In addition, they were born more often to mothers with asthma and hypertension and to parents who smoked during pregnancy. Furthermore, their mothers had received more infertility treatments before pregnancy compared with the controls. Prevalence of asthma was highest among lower white-collar workers and lowest among entrepreneurs (Table 1).

**Table 1:** **Basic maternal and perinatal characteristics of the study population in relation to asthma ever**

**Characteristics**	**No asthma N(%)**	**Asthma N(%)**	***p*** **value**
**Maternal age, years**			
<20	1246(3.3)	69(2.7)	
20-29	19 613(52.3)	1366(54.2)	
30-39	15 544(41.3)	1015(40.3)	
≥40	1197(3.2)	68(2.7)	0.083
**Maternal parity, primiparous**	15 359(40.7)	967(38.4)	0.016
**Maternal asthma**	1016(2.7)	113(4.5)	<0.001
**Maternal gestational diabetes mellitus**	2619(7.0)	143(5.7)	0.014
**Maternal hypertension**	716(1.9)	74(2.9)	<0.001
**Assisted reproduction technology, index pregnancy** ^1^	1514(4.0)	142(5.6)	<0.001
Pre-pregnancy BMI*, kg/m ^2^			
<25	25 675(71.4)	1702(70.6)	
25-29.9	7105(19.8)	480(19.9)	
≥30	3182(8.8)	228(9.5)	0.557
**Marital status**			
Unmarried	3938(10.5)	274(10.9)	
Married or cohabiting	33 662(89.5)	2244(89.1)	0.518
**Socioeconomic status**			
Upper white-collar worker	5375(14.3)	359(14.3)	
Lower white-collar worker	13 790(36.7)	973(38.6)	
Blue-collar worker	7528(20.0)	477(18.9)	
Entrepreneurs	1115(3.0)	52(2.1)	
Other	7582(20.2)	543(21.6)	
Missing	2210(5.9)	114(4.5)	0.001
Parental smoking**			
Non-smoking parents	19 215(55.2)	1240(52.3)	
Maternal/paternal smoking	8992(25.8)	633(26.7)	
Both smoking parents	6620(19.0)	498(21.0)	0.013
**Mode of delivery**			
Vaginal	31 288(83.2)	2000(79.4)	
Cesarean delivery	6312(16.8)	518(20.6)	<0.001
**Sex of child, boy**	18 940(50.4)	1521(60.4)	<0.001
**Gestational age at birth, weeks**			
<28	129(0.3)	50(2.0)	
28-32	341(0.9)	80(3.2)	
33-36	1579(4.2)	149(5.9)	
37-40	26 253(69.8)	1724(68.5)	
≥41	9298(24.7)	515(20.5)	<0.001
**Birth weight <2500 grams**	1482(3.9)	221(8.8)	<0.001
**Small for gestational age (SGA)**	13569(9.5)	263(10.4)	0.117
**Asthma prevalence by age group, years**			
1-6	13 251(35.2)	445(17.7)	
7-12	10 390(27.6)	945(37.5)	
≥13	13 959(37.1)	1128(44.8)	<0.001
**Mean age at the onset of asthma, years (±SD)**		4.2(3.5)	
**Total study population**	37600(93.7)	2518(6.3)	

^1^Assisted reproduction technology before index pregnancy including in vitro fertilization, intra-cytoplasmic sperm injection, insemination and ovulation induction by clomifene citrate and other medicines.

^2^BMI: kilograms/square meters.

*Body mass index, data missing 1746 (4.4%) cases.

**Data missing 2920 (7.3%) cases.

We further evaluated maternal and prenatal differences between the various socioeconomic groups (Tables 2 and 3). As expected, Table 2 shows that all but one of the evaluated maternal characteristics was significantly associated with socioeconomic factors. Upper white-collar workers were older, more often married, with lower BMI, with a high rate of fertility problems and with a low number of pregnancies and artificial abortions compared with the other SES groups. Over 60% of the blue-collar workers were less than 30 years of age, 10.6% were heavily overweight, in 26.45 of cases both parents smoked and they had more previous artificial abortions than the other groups.

**Table 2:** **Maternal background characteristics by socioeconomic status (SES)**

	**Total n(%)**	**Upper white-collar worker n(%)**	**Lower white-collar worker n(%)**	**Blue-collar worker n(%)**	**Other n(%)**	**Missing n(%)**	**p value**
Maternal age, years							
<20	1315(3.3)	10(0.2)	125(0.8)	196(2.4)	788(8.5)	196(8.4)	
20-29	20 979(52.3)	2087(36.4)	7426(50.3)	4629(57.8)	5658(60.9)	1179(50.7)	
30-39	16 559(41.3)	3406(59.4)	6728(45.6)	2942(36.8)	2617(28.2)	866(37.3)	
≥40	1265(3.2)	231(4.0)	484(3.3)	238(3.0)	229(2.5)	83(3.6)	<0.001
Asthma	1129(2.8)	136(2.4)	438(3.0)	172(2.1)	285(3.1)	98(4.2)	<0.001
Hypertension	790(2.0)	94(1.6)	325(2.2)	158(2.0)	169(1.8)	44(1.9)	0.074
ART^1^	1656(4.1)	327(5.7)	717(4.9)	304(3.8)	244(2.6)	64(2.8)	<0.001
Infertility (own opinion)	2290(5.7)	489(8.5)	971(6.6)	428(5.3)	316(3.4)	86(3.7)	<0.001
Pre-pregnancy BMI^2^, kg/m^2^							
<25	27 377(71.3)	4232(76.7)	10 140(71.2)	5180(67.4)	6439(72.1)	1386(69.2)	
25-29.9	7 585(19.8)	975(17.7)	2871(20.2)	1690(22.0)	1659(22.0)	390(19.5)	
≥30	3 410(8.9)	310(5.6)	1227(8.6)	815(10.6)	831(9.3)	227(11.3)	<0.001
Marital status							
Unmarried	4 212(10.5)	360(6.3)	1364(9.2)	1038(13.0)	1110(11.9)	340(14.6)	
Married or cohabiting	35 906(89.5)	5374(93.7)	13 399(90.8)	6967(87.0)	8182(88.1)	1984(85.4)	<0.001
Mean number of deliveries^3^	1.0(1.3)	1.0(1.2)	1.0(1.1)	1.0(1.2)	1.3(1.6)	0.9(1.3)	<0.001
Mean number of pregnancies^3^	1.4(1.6)	1.3(1.5)	1.4(1.4)	1.4(1.5)	1.7(2.0)	1.4(1.6)	<0.001
Mean numbers of miscarriages^3^	0.2(0.6)	0.2(0.6)	0.2(0.5)	0.2(0.5)	0.2(0.6)	0.2(0.6)	0.004
Mean number of artificial abortions^3^	0.1(0.4)	0.1(0.3)	0.1(0.4)	0.2(0.5)	0.1(0.4)	0.2(0.4)	0.001
Surgically scarred urerus	3590(8.9)	513(8.9)	1325(9.0)	776(9.7)	831(8.9)	145(6.2)	<0.001
**Total**	40 118(100)	5734(14.3)	14 763(36.8)	8005(20.0)	9292(23.2)	2324(5.8)	

^1^Assisted reproduction technology before index pregnancy including in vitro fertilization, intracytoplasmic sperm injection, insemination and ovulation induction by clomifene and other medizines.

^2^Body mass index, data missing in 1746 (4.4%) cases.

^3^Data are presented as mean (±SD).

**Table 3:** **Prenatal characteristics by maternal socioeconomic status (SES)**

	**Total**	**Upper white-collar worker n(%)**	**Lower white-collar worker n(%)**	**Blue-collar worker n(%)**	**Other n(%)**	**Missing n(%)**	**p value**
**Primiparous**	16 326(40.7)	2346 (40.9)	6006(40.7)	3221(40.2)	3572(38.4)	1181(50.8)	
**Multiparous**	23 792(59.5)	3388(59.1)	8757(59.3)	4784(59.8)	5720(61.6)	1143(49.2)	<0.001
**Gestational diabetes mellitus**	2762(6.9)	350(6.1)	1086(7.4)	565(7.1)	540(5.8)	221(9.5)	<0.001
**Pre-eclampsia**	1466(3.7)	175(3.1)	593(4.0)	325(4.1)	297(3.2)	76(3.3)	<0.001
Fear of childbirth*	625(1.6)	98(1.7)	242(1.6)	83(1.0)	152(1.6)	50(2.2)	<0.001
Parental smoking**							
Non-smoking	20 455(55.0)	3919(72.5)	7946(57.3)	3352(44.5)	4382(51.0)	856(47.6)	
Maternal/paternal smoking	9625(25.9)	1060(19.6)	3716(26.8)	2193(29.1)	2197(25.5)	459(25.5)	
Smoking	7118(19.1)	425(7.9)	2195(15.8)	1993(26.4)	2020(23.5)	485(26.9)	<0.001
**Mode of delivery**							
Vaginal	33 280(83.0)	4685(81.7)	12 138(82.2)	6629(82.8)	7929(85.3)	1899(82.0)	
Elective cesarean	3176(7.9)	527(9.2)	1226(8.3)	599(7.5)	624(6.7)	200(8.6)	
Cesarean section during labour	3654(9.1)	522(9.1)	1399(9.5)	777(9.7)	739(8.0)	217(9.4)	<0.001
**Sex of child, Boy**	20 461(51.0)	2940(51.3)	7550(51.1)	3995(49.9)	4753(51.2)	1223(52.6)	0.155
**Gestatonal age at birth, weeks**							
<37	2325(5.5)	290(5.1)	854(5.8)	491(6.1)	515(5.5)	175(7.5)	
37-40	27 977(69.7)	4103(71.6)	10 334(70.0)	5456(68.2)	6487(69.8)	1597(68.7)	
≥41	9813(24.5)	1340(23.4)	3575(24.2)	2056(25.7)	2290(24.6)	552(23.8)	<0.001
Mean birth weight, grams***	3517(589)	3541(560)	3525(587)	3496(602)	3523(584)	3457(642)	0.001
**Apgar score <7 at 5 min**	689(1.7)	91(1.6)	246(1.7)	144(1.8)	152(1.6)	56(2.4)	0.089
**Care in Intensive Care Unit(NICU)**	3466(8.6)	517(9.0)	1298(8.8)	701(8.8)	729(7.8)	221(9.5)	0.024
**Placental abruption**	257(0.6)	38(0.7)	74(0.5)	71(0.9)	54(0.6)	20(0.9)	0.006
**Birth weight <2500 g**	1703(4.2)	193(3.4)	606(4.1)	375(4.7)	381(4.1)	148(6.4)	<0.001
**Small for gestational age**	3832(9.6)	500(8.7)	1316(8.9)	882(11.0)	867(9.3)	267(11.6)	<0.001
**Total**	40 118(100)	5734(14.3)	14 763 (36.8)	8005 (20.0)	9292(23.2)	2324 (5.8)	

*Data available years in 1996-2007.

**Data available in 37 198 cases.

***Data are presented as mean (SD).

Maternal socioeconomic factors were also associated very strongly with most of the studied prenatal factors (Table 3). Upper white-collar workers more often had fear of childbirth, but less preeclampsia and they smoked less during pregnancy. They delivered more often at term by elective cesarean delivery than their counterparts with lower SES. Their neonates had the highest birth weights, the lowest incidence of being SGA with low birth weight, but interestingly they most often needed neonatal intensive care unit treatment after birth. In contrast, preterm birth, preeclampsia, cesarean delivery during labor and birth after 41 weeks of gestation, with lower Apgar scores, were more common among blue-collar workers compared with the other SES groups.

In univariate analysis, children in the “other” group (including housewives, the unemployed and students) had the highest rate of asthma (6.4%), but no clear differences were detected between groups (e.g. upper white-collar vs. blue-collar workers; 6.3% vs. 6.0%). In this analysis, the children of entrepreneurs had the lowest prevalence of asthma (4.5%), but since entrepreneurs constituted only 2.9% of all study women, they were not analyzed separately but included in the “other” group. In adjusted analysis, no significant differences were detected in the risk of asthma among offspring in relation to maternal SES at the time of pregnancy (Table 4). Belonging to the “missing” group (maternal SES not defined) was associated with a protective effect against asthma (aOR 0.42, 95% CI 0.32–0.54), reflecting the fact that the group was very heterogeneous.

**Table 4:** **Risk of asthma ever among offspring by maternal socioeconomic status (SES)**

**SES**	**Total n(%)**	**Asthma n(%)**	**OR(95% CI)**	**p value**	aOR(95% Cl)*	**p value**
**Upper white-collar worker**	5734(14.3)	359(6.3)	1		1	
**Lower white-collar worker**	14 763(36.8)	973(6.6)	1.06(0.93-1.20)	0.390	1.02(0.89-1.16)	0.816
**Blue-collar worker**	8 005(20.0)	477(6.0)	0.95(0.82-1.09)	0.465	1.01(0.86-1.17)	0.950
**Other**	9292(23.2)	595(6.4)	1.02(0.90-1.17)	0.728	1.02(0.88-1.18)	0.776
**Missing**	2324(5.8)	114(4.9)	0.77(0.62-0.96)	0.019	0.42(0.32-0.54)	<0.001
**Total**	40118(100)	2518(6.3)				

*p* value in adjusted analysis obtained from the trend test(Wald) in logistic regression models.

*Adjusted for maternal factors (asthma,hypertension, parity and ART), parental smoking and neonatal factors (sex, gestational age at birth, mode of delivery and the onset of asthma or among controls the followup time).

## Discussion

The overall prevalence of asthma among children was 6.3%, varying between 4.9–6.6% in the different maternal socioeconomic groups. We detected no significant associations in univariate or in multivariate analysis between maternal SES and asthma after defining four SES groups (upper-white collar workers, lower white-collar workers, blue-collar workers, and “other”). Our results are in line with those of previously published studies and support the concept that the etiology of asthma is multifactorial [15]. However, in subgroup analysis the children of entrepreneurs had the lowest prevalence of asthma (4.5%). Since this professional group represented only 2.9% of the pregnant women, the group was underpowered to be analyzed individually in multivariate analysis, but it was included in the “other” group. In our area, interestingly, female entrepreneurs were most commonly farmers, and according to earlier reports, a low prevalence of asthma among farmers’ children is an expected finding.

We found that mothers with lower SES (blue-collar and others) were often multiparous women delivering vaginally (82.8–85.3%) at term or after (93.9–94.4%), factors known to be protective against asthma [1,3,16]. Ekblad et al. (2013) reported that smoking was more likely among teenagers in single-mother and lower-SES groups [17]. We found similar results, since up to a fourth of parents smoked during pregnancy in the lower-SES groups. They were also twice as often unmarried compared with those in the higher-SES groups (13%; 6.3%) and over 60% of mothers in the lower-SES groups were under 30 years of age and among the lowest (“other”) group 8.5% were under 20 years of age. Prenatal protective factors, such as lower infertility, maternal multiparity and vaginal delivery might be reasons for the lower prevalence of asthma among lower-SES groups, since smoking, known to be a significant risk factor of asthma [1,18], was more prevalent among these lower-SES groups compared with lower white-collar workers, where asthma was most common (6.6%). Inclusion of entrepreneurs, consisting mostly of agricultural producers with a low risk of offspring asthma, diminished asthma prevalence as a net effect among children in the “other” group.

In our large register-based study, some obstetric disparities were found between the different socioeconomic groups. We showed that elective cesarean delivery was most frequent in the highest SES group and non-planned cesarean delivery in blue-collar workers. A total of 26.4% of blue-collar mothers had smoked, predisposing themselves to SGA infants and placental abruption (prevalences highest at 11.0% and 0.9% in this SES group) and to preeclampsia, which in turn predisposed them to non-elective cesarean delivery. Advanced maternal age, preterm birth and some delivery characteristics, such as placental abruption, preeclampsia and SGA infants are strongly associated with giving birth by cesarean section [8,9,19].

We found no convincing evidence of a direct association between maternal SES and childhood asthma. Finnish public health services are available to all citizens and private services are also partly covered by the Social Insurance Institution. Furthermore, diagnosis and treatment of asthma is similar for all children. To be eligible to receive reimbursement payments under the Special Refund Categories the patient must obtain a certificate provided as a semi-structured form from a doctor and disease must be based on clinical diagnosis and criteria (ICD-10). In the case of pediatric asthma a certificate is usually issued by a pediatrician in order to confirm the nature of the disease and the need for long-term medication. Minor differences might be a result of the nature of access to care, or the quality of treatment of asthma in different SES groups [20]. Parental smoking during pregnancy was found to be a strong independent factor of asthma among the children of lower white-collar workers and among these smoking families children might also be likely to grow up in a smoking household. An interesting finding was that the prevalence of childhood asthma among blue-collar workers was lowest (6.0%), even though smoking was most prevalent in that group. This may be related to reduced access to medical care in this group.

The most important strength of this study was that a large amount of data was collected from a single university hospital with uniform data-collection criteria and this therefore offered a comprehensive and reliable view of birth characteristics, perinatal outcome and socioeconomic factors. On the other hand, the register was produced mainly for administrative and statistical purposes rather than for research and therefore it might have included errors, and the percentage of missing SES values was 5.8%, which is quite low number and possibly included younger women and immigrants taking care of their children at home and thus decreasing risk of asthma. National registers in Finland have been shown to be of high quality as regards validity and accuracy [21] and thus we obtained valuable and reliable information on pharmaceutical data. Information on SES was based solely on maternal occupation at the time of birth and information on household income and fathers’ socioeconomic position was not available. However, in Finland income is known to correlate with occupation and therefore by proxy is sufficient to indicator for studies on socioeconomic health differences in perinatal period [22-24].

A limitation of our study was that ethnic/racial disparities in different countries are known to influence both SES and asthma [1,25]. In 2009 ethnic minorities constituted only 4% of the whole Finnish population [26], being a minority in our study population. We also lack information on parental smoking habits after birth and mothers’ breastfeeding as additional confounding factors, also known to be risk factors of asthma. Furthermore, information on environmental risk factors such as day-care attendance, respiratory infections during childhood and pollution were not available, and mothers from lower-SES groups are known to be less likely to breastfeed their children and take them to day-care facilities [1,3,27].

## Conclusion

A direct association between SES and childhood asthma was not found in our Finnish population, but SES indirectly affects childhood asthma via adverse prenatal exposures [14,17,28]. Disparities are the result of the existence of differences in health behavior and healthcare use during pregnancy, especially as regards women from low social classes (i.e., unemployed or poorly educated). Such inequalities should be examined in public health and other public services at a wider level, to ensure equal opportunity of use.

## Abbreviations

SES: Socioeconomic status
SGA: Small for gestational age
aOR: Adjusted odds ratio
Cl: Confidence interval
